# Increased Temperature Facilitates Adeno-Associated Virus Vector Transduction of Colorectal Cancer Cell Lines in a Manner Dependent on Heat Shock Protein Signature

**DOI:** 10.1155/2020/9107140

**Published:** 2020-02-08

**Authors:** Alicja Bieńkowska, Weronika Kuźmicka, Olga Ciepiela, Justyn Ochocki, Maciej Małecki

**Affiliations:** ^1^Department of Applied Pharmacy, Faculty of Pharmacy with Laboratory Medicine, Medical University of Warsaw, 1 Banacha Street, 02-097 Warsaw, Poland; ^2^Department of Laboratory Medicine and Clinical Immunology of Developmental Age, Faculty of Medicine, Medical University of Warsaw, 63a Żwirki i Wigury Street, 02-091 Warsaw, Poland; ^3^Postgraduate School of Molecular Medicine, Medical University of Warsaw, 61 Żwirki i Wigury Street, 02-091 Warsaw, Poland; ^4^Department of Bioinorganic Chemistry, Medical University, 1 Muszynskiego Street, 90-150 Lodz, Poland

## Abstract

Colorectal cancer (CRC) is one of the most common cancers in human population. A great achievement in the treatment of CRC was the introduction of targeted biological drugs and solutions of chemotherapy, combined with hyperthermia. Cytoreductive surgery and HIPEC (hyperthermic intraperitoneal chemotherapy) extends the patients' survival with CRC. Recently, gene therapy approaches are also postulated. The studies indicate the possibility of enhancing the gene transfer to cells by recombinant adeno-associated vectors (rAAV) at hyperthermia. The rAAV vectors arouse a lot of attention in the field of cancer treatment due to many advantages. In this study, the effect of elevated temperature on the transduction efficiency of rAAV vectors on CRC cells with different origin and gene profile was examined. The effect of heat shock on the penetration of rAAV vectors into CRC cells in relation with the expression of HSP and AAV receptor genes was tested. It was found that the examined cells under hyperthermia (43°C, 1 h) are transduced at a higher level than in normal conditions (37°C). The results also indicate that studied RKO, HT-29, and LS411N cell lines express HSP genes at different levels under both 37°C and 43°C. Moreover, the results showed that the expression of AAV receptors increases in response to elevated temperature. The study suggests that increased rAAV transfer to CRC can be achieved under elevated temperature conditions. The obtained results provide information relevant to the design of new solutions in CRC therapy based on the combination of hyperthermia, chemotherapy, and gene therapy.

## 1. Introduction

Colorectal cancer (CRC) is one of the most common cancers in human population [1]. According to data from 2018, CRC is the second cause of oncology patient deaths in the world. The incidence and mortality rate of CRC in 2018 was above 1.8 million and nearly 0.9 million patients, respectively. Epidemiological studies indicate a continuous increase in cases of CRC [1]. At the same time, there is a dynamic progress in the field of explaining molecular mechanisms of CRC growth, defining predictors, as well as developing and implementing new drugs and therapeutic programs for patients [2–5]. The clinical centers especially administer drug programs based on fluoropyrimidine, leucovorin and oxaliplatin (5-fluorouracil-leucovorin-oxaliplatin, FOLFOX program) [6]. A great achievement was the introduction of targeted biological drugs to inactivate the key receptor/signaling proteins in CRC. The representative example is the presence in the clinics of cetuximab (anti-EGFR monoclonal antibody) and bevacizumab (anti-VEGF monoclonal antibody). These drugs, however, have functional limitations and very often numerous side effects. For example, cetuximab is most of all used in patients with a nonmutated (wild type) KRAS gene [7, 8]. Moreover, in the CRC treatment area, solutions of chemotherapy combined with hyperthermia are introduced. It is indicated that the elevated temperature increases the therapeutic activity of the used cytotoxic drugs, especially in cases of peritoneal carcinomatosis [9, 10]. Increasing the permeability of tumor cell membranes, blood vessels and changes in the response of the immune system are emphasised among the postulated antineoplastic mechanisms of hyperthermia [11]. The importance of heat shock proteins (HSP) is highlighted in the response of tumor cells to hyperthermia and cytostatics are emphasized [12, 13]. Hyperthermia protocols include local application of elevated temperature or heating the whole body. Currently, nanotechnology proposes different ways of generating the phenomenon of hyperthermia, including laser, microwaves, radiofrequency, and ultrasound sources [14]. Great importance is attached to the development of the HIPEC strategy (hyperthermic intraperitoneal chemotherapy) [15]. Cytoreductive surgery and HIPEC extends the survival of patients with CRC [16, 17]. The results of a meta-analysis published recently by Desidero et al. including papers published in the last 30 years, showed that HIPEC significantly increases the survival time of patients with gastric cancers [18]. The number of new HIPEC clinical protocols in the field of CRC is growing [19, 20]. The possibilities of using the oncology hyperthermia strategy in increasing the effectiveness of cancer gene therapy based on recombinant adeno-associated vectors (rAAV) are also indicated [21–23]. Hyperthermia may induce both an increase in the expression of transgenes under the control of temperature-dependent inducible promoters [24, 25], and increase the vector transduction efficiency in a mechanism dependent on the HSP expression [22, 26].

Advances in understanding of CRC biology enable the development of ultra-modern drugs and therapies, which include gene transfer/correction strategies. Gene therapy is a promising proposition in cancer treatment. According to scientists, gene therapy based on the CRISPR-Cas9 method (localization and repair of damaged genes), lenivirus and adeno-associated virus vectors will play a key role in science and medicine in the coming years [27, 28]. Currently, cancer gene therapy strategies especially include suicide therapy, inhibition of angiogenesis, apoptosis stimulation, and modulation of immune response [29]. The development of gene transfer methods depends directly on progress in vectorology. Vectors based on rAAV arouse a lot of attention in the field of cancer treatment due to lack of pathogenicity, transduction of dividing and nondividing cells, selective tissue tropism, stable and long-term expression of the transgene [30–32]. In addition, it is suggested that rAAV have a natural tropism for colon cancer cells [33]. The current challenge in the field of rAAV vectors is to increase the efficiency of transduction through multiplying the applied dose of rAAV (MOI), using rAAV serotypes with defined organ tropism, chemical transduction modulators, e.g. proteasome inhibitors, and incorporating physicochemical procedures into the transduction [32]. Attention is drawn to research indicating the possibility of enhancing the transfer of rAAV genes by hyperthermia [21–23]. Due to the presence of hyperthermia in the treatment procedures of oncological patients, the design of clinical trials in cancer gene therapy should consider the possibility of increasing the efficiency of rAAV transduction through heat shock. Moreover, the observed gene signatures of cancer cells exposed to hyperthermia facilitates the introduction to oncological clinics of new therapeutic solutions, such as cancer gene therapy based on rAAV vectors.

The present study investigates the effect of elevated temperature (conditions imitating the oncological hyperthermia) on the transduction of rAAV vectors to CRC with different origin and gene profile. The effect of heat shock on the penetration of rAAV vectors into CRC cells in relation to the expression of key genes determining carcinogenicity and rAAV transduction was investigated. Emphasis was placed on the expression of HSP genes. The work provides essential information to the design of new solutions in CRC therapy based on the combination of hyperthermia, chemotherapy, and gene therapy.

## 2. Materials and Methods

### 2.1. Cell Lines

The following human colon cancer cell lines, part of Colon Cancer Panel 2, BRAF (ATCC® TCP-1007™, American Type Culture Collection, Manassas, VA, USA) were included in the present study: HT-29 (ATCC® HTB-38™), LS411N (ATCC® CRL-2159™), RKO (ATCC® CRL-2577™), and CCD-18Co (ATCC® CRL-1459™) human colon fibroblast. HT-29 were cultured in McCoy's 5a Medium Modified (Sigma-Aldrich, St. Louis, MO, USA), LS411N–RPMI-1640 Medium (Gibco, Thermo Fisher Scientific, Waltham, MA, USA), RKO–Dulbecco's Modified Eagle Medium (DMEM; Gibco, Thermo Fisher Scientific), CCD-18Co–Minimum Essential Medium (MEM; Gibco, Thermo Fisher Scientific). The media were supplemented with 10% FBS (Gibco, Thermo Fisher Scientific) and 1% Antibiotic–Antimycotic (Gibco, Thermo Fisher Scientific). The cells were maintained at 37°C in a humidified 5% CO_2 _atmosphere.

### 2.2. rAAV Transduction at Hyperthermia

Cells were seeded at the density of 6.5 × 10^4^ for HT-29 and LS411N, 3.5 × 10^4^ for RKO and 10.0 × 10^4^ for CCD-18Co per well in six-well plates (Nunc, Thermo Fisher Scientific) in dedicated medium. After 24 h incubation at a normal culture condition, cell lines were transduced. The recombinant AAV vector AAV/DJ-CAG-GFP (Cat. No. 7078; Vector Biolabs, Malvern, PA, USA) was used with the multiplicity of infection (MOI) of 4 × 10^4^ genome copies. The AAV/DJ is a mosaic serotype made with genetic engineering methods from eight wild types of AAV serotypes, including AAV2, 4, 5, 8, 9, avian, bovine, and goat AAV. The vector expresses eGFP (green fluorescent protein) under the control of CAG promoter, which is a hybrid of the cytomegalovirus (CMV) early enhancer element and chicken beta-actin promoter [34]. Before transduction, the cell culture media were replaced with dedicated media heated up to 37°C or 43°C enriched with 2% FBS. Transduction was performed in two variants. The first option consisted in incubating cells for 1 h at 43°C, then rAAV was introduced to the media. The second variant began with adding the vectors to the media; then the plate was shaken at 300 rpm in a 5 × 4 minute system, with alternate temperature changes starting from 43°C for 4 min, then changing to 37°C for 4 min, and back to 43°C for 4 min, etc. (Eppendorf ThermoMixer® C, Eppendorf AG, Hamburg, Germany). In addition, a transduction was performed at 37°C without shaking (control to hyperthermia) and with shaking at 300 rpm for 20 min (to verify the influence of shaking on the transduction efficiency). Subsequently the cells were moved to 37°C with a humidified 5% CO_2 _atmosphere. Control samples (not subjected to transduction) for each temperature/transduction condition were performed. After 48 h and 96 h of transduction, the cell culture media were replaced with dedicated fresh media enriched with 2% FBS. Finally, on the sixth day after transduction the cells were harvested.

### 2.3. Measurement of Transduction Efficiency

The inverted fluorescence microscope (Olympus IX53, Olympus, Tokyo, Japan) with the pE-300^white^(CoolLED, Andover, MA, USA) illumination system was used to obtain the images of cells expressing Green Fluorescent Protein (GFP). Pictures of nontransduced (in BF) and transduced (in FITC) cells were taken at 10x magnification. The percentage of GFP positive cells was determined with the use of The Countess II FL Automated Cell Counter (Invitrogen, Thermo Fisher Scientific) with EVOS Light Cube–GFP (470/22 nm Excitation; 510/42 nm Emission) and flow cytometry (LSR Fortessa, Becton Dickinson, Franklin Lakes, NJ, USA). Briefly, to determine cell viability and transduction efficiency by FACS, 2 × 10^6^/mL cells were washed, centrifuged, resuspended in PBS and analyzed. 7-Aminoactinomycin D (7-AAD) was used to determine the number of dead cells (PerCP-Cy 5.5 detector). Percentage of transduced cells was analyzed based on the level of green fluorescent protein expression (FITC detector).

All GFP+ cell measurements were performed on the 7th day of the experiment, then the cells were harvested for DNA isolation to analyze the transduction efficiency by qPCR.

### 2.4. Real-Time PCR for Determination of AAV Genome Copy Number

Total DNA was isolated using the High Pure Viral Nucleic Acid Kit (Roche Life Science, Mannheim, Germany). To determine the rAAV genome copy number, TaqMan assays were designed using a probe and primers for the ITR region. The TaqMan probe (5′-CACTCCCTCTCTGCGCGCTCG-3′) featured 6-FAM and TAMRA. The forward primer was 5′-GGAACCCCTAGTGATGGAGTT-3′ and the reverse primer was 5′-CGGCCTCAGTGAGCGA-3′ [35]. Standard curve was created using plasmid DNA pAAV-hrGFP (Part No. 240074; Agilent Technologies). Total volume of qPCR reactions was 10 *µ*l, contained 50 ng DNA and ran under the following conditions: 50°C for 2 minutes, 95°C for 10 minutes, 40 cycles of 95°C for 15 seconds, and 60°C for 60 s. Real-time PCR was performed in StepOnePlus™ Real-Time PCR System (Applied Biosystems, Thermo Fisher Scientific). The rAAV genome copy number was normalized as viral genome copy number/50 *µ*g of total genomic DNA.

### 2.5. Reverse Transcription PCR and Real-Time PCR

Total RNA was isolated [36] using the TRI Reagent (Sigma-Aldrich, St. Louis, MO, USA) to test gene expression. The contaminating DNA in RNA samples was removed with the use of DNA-free™ DNA Removal Kit (Invitrogen, Thermo Fisher Scientific). The High Capacity RNA-to-cDNA kit (Applied Biosystems, Thermo Fisher Scientific) was used to synthesise single-stranded cDNA.

The expression of the extracellular AAV transmission genes was examined with the use of the following TaqMan Assays (assay ID, Thermo Fisher Scientific): AAVR (Hs00967343_m1), HSPG1 (Hs01081432_m1), HSPG2 (Hs01078536_m1), and ACTB (Hs01060665_g1) as endogenous control. The expressions were measured after 24 h from exposure to 37°C and 43°C for 1 h. Samples of CCD-18Co maintained at 37°C were selected as a reference sample in the ΔΔCt method [37].

The TaqMan Array 96–Well FAST Plate Human Heat Shock Proteins (Cat. No. 4418733; Applied Biosystems, Thermo Fisher Scientific) was used to examine the expression of HSP in studied cell lines, 24 h after exposure to the hyperthermia condition. As reference samples were selected results for cells maintained at 37°C and used for the comparison of constitutive levels of HSP in colon cancer cell lines and CCD-18Co.

Gene expression levels of target genes were normalized to the level of housekeeping genes encoding: *β*-actin (ACTB) for AAV transmission genes, glyceraldehyde-3-phosphate dehydrogenase (GAPDH) for HSP. All qPCR reactions were performed based on manufacturer instructions in the StepOnePlus™ Real-Time PCR System. The Expression Suite Software v1.1 (Thermo Fisher Scientific) was used to calculate relative gene expression levels by the ΔΔCt method [37].

The STRING database was used to prepare string diagrams presenting physical and/or functional interactions between proteins encoded by examined transcripts [38].

### 2.6. Statistical Analysis

The results were expressed as mean and standard deviation. The statistical significance of differences between mean values of compared samples was calculated using the one-way analysis of variance (ANOVA, *α* = 0.05) with a post-hoc Tukey test (*α* = 0.05) using GraphPad Prism 7 (GraphPad Software, La Jolla, CA, USA). Differences were considered significant if *p* < 0.05. The statistical significance is marked as asterisks (^∗^*p* < 0.05; ^∗∗^*p* < 0.01; ^∗∗∗^*p* < 0.001, ^∗∗∗∗^*p* < 0.0001) or as carets (^*p* < 0.05; ^^*p* < 0.01; ^^^*p* < 0.001; ^^^^*p* < 0.0001).

## 3. Results

### 3.1. rAAV Transduction of Colorectal Cancer Cells at Hyperthermia Conditions

**Figure 2:**
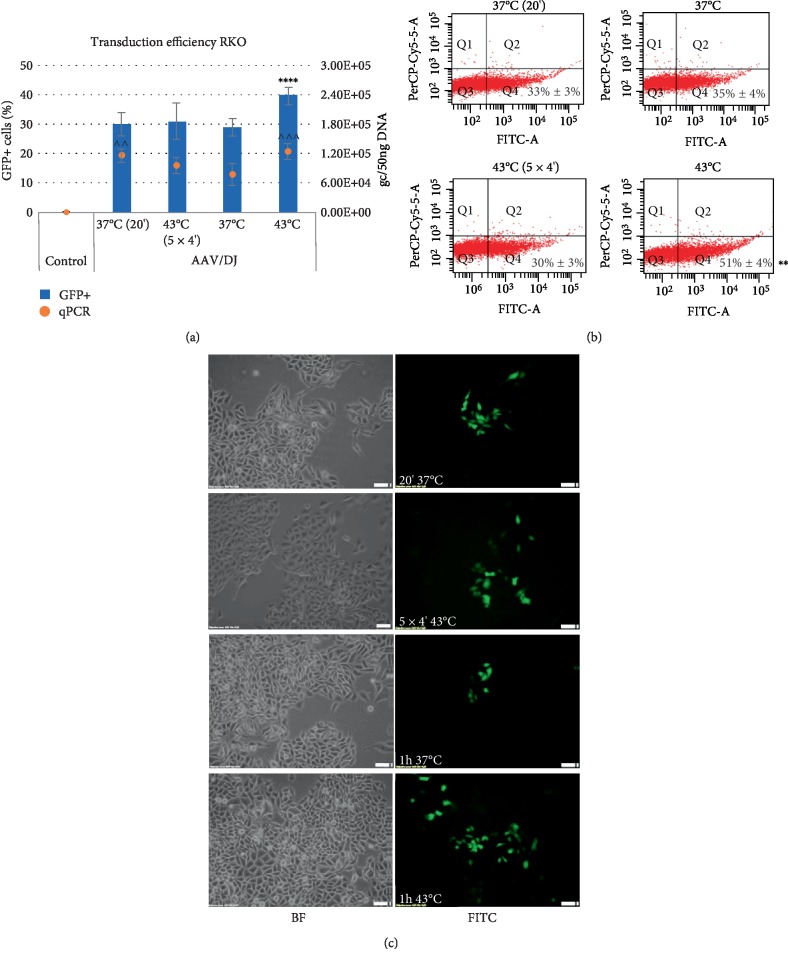
Transduction efficiency of RKO cells. The studied cells were transduced with the rAAV/DJ vector at hyperthermia conditions (as described at M&M panel). The GFP (Green Fluorescent Protein) positive (+) cells were measured by the Countess II FL Automated Cell Counter and the qPCR method (panel (a)), flow cytometry (panel (b)), and visualized with an inverted fluorescence microscope (panel (c)). For panel (b) the Q1 and Q2 quadrants represents death cells (7-AAD positive staining). The Q3 and Q4 quadrants represents live cells (7-AAD negative staining). The GFP+ cells are located in Q2 and Q4 quadrants. Images of non transduced (BF) cells and transduced cells (FITC) were performed at 10x magnification (panel (c)). Statistically significant differences were analyzed between cells transduced at 37°C and cells transduced at hyperthermia conditions. Statistical significance for results of GFP+ cells were marked as asterisks (^∗∗^*p* < 0.01; ^∗∗∗∗^*p* < 0.0001) and for results of rAAV genome copies (gc) were marked as carets (^^*p* < 0.01; ^^^*p* < 0.001).

**Figure 3:**
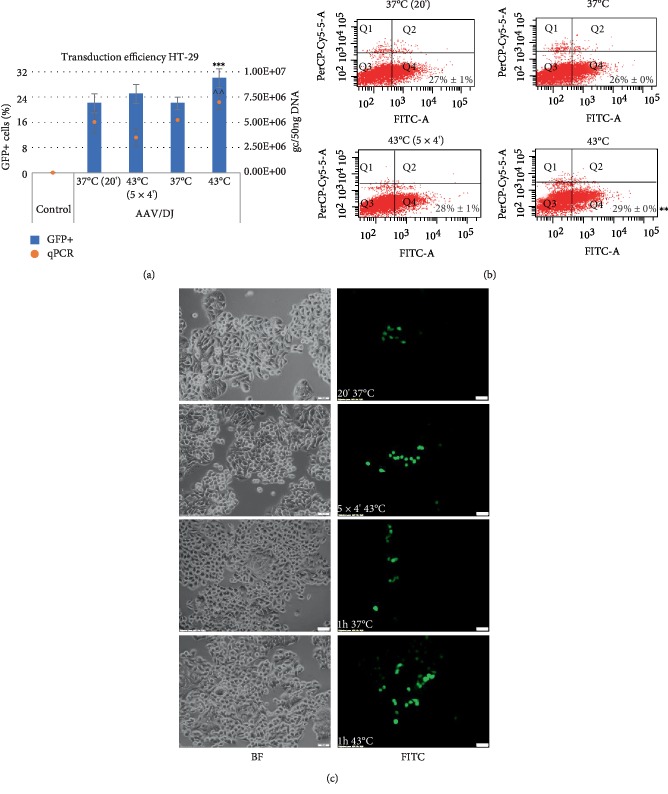
Transduction efficiency of HT-29 cells. The studied cells were transduced with the rAAV/DJ vector at hyperthermia conditions (as described at M&M panel). The GFP (Green Fluorescent Protein) positive (+) cells were measured by the Countess II FL Automated Cell Counter and the qPCR method (panel (a)), flow cytometry (panel (b)), and visualized with an inverted fluorescence microscope (panel (c)). For panel (b) the Q1 and Q2 quadrants represents death cells (7-AAD positive staining). The Q3 and Q4 quadrants represents live cells (7-AAD negative staining). The GFP+ cells are located in Q2 and Q4 quadrants. Images of non transduced (BF) cells and transduced cells (FITC) were performed at 10x magnification (panel (c)). Statistically significant differences were analyzed between cells transduced at 37°C and cells transduced at hyperthermia conditions. Statistical significance for results of GFP+ cells were marked as asterisks (^∗∗^*p* < 0.01; ^∗∗∗^*p* < 0.001) and for results of rAAV genome copies (gc) were marked as carets (^^*p* < 0.01).

**Figure 4:**
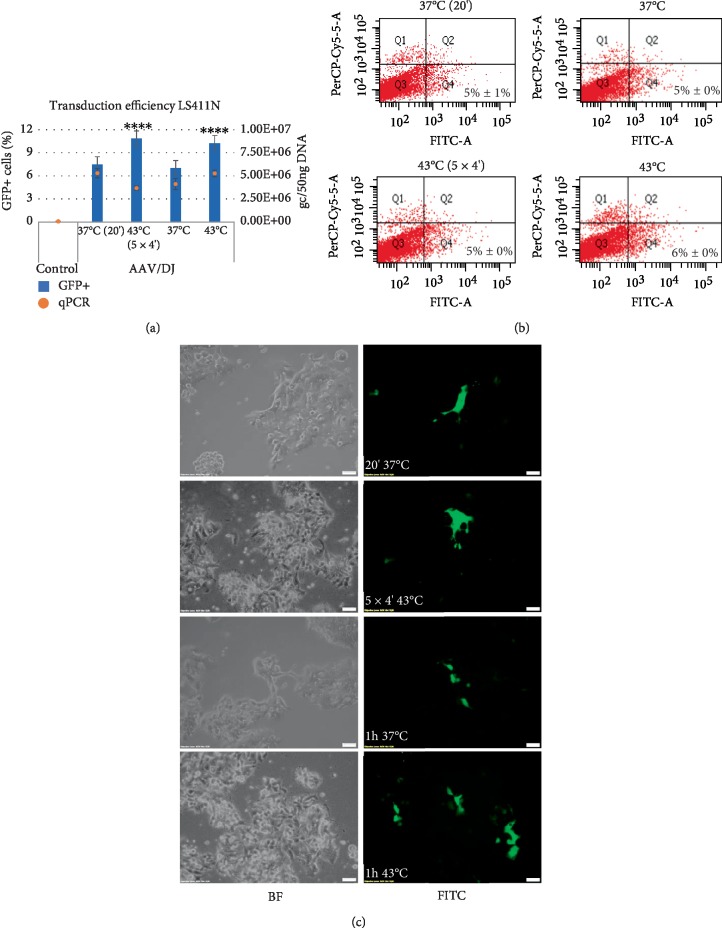
Transduction efficiency of LS411N cells. The studied cells were transduced with the rAAV/DJ vector at hyperthermia conditions (as described at M&M panel). The GFP (Green Fluorescent Protein) positive (+) cells were measured by the Countess II FL Automated Cell Counter and the qPCR method (panel (a)), flow cytometry (panel (b)), and visualized with an inverted fluorescence microscope (panel (c)). For panel (b) the Q1 and Q2 quadrants represents death cells (7-AAD positive staining). The Q3 and Q4 quadrants represents live cells (7-AAD negative staining). The GFP+ cells are located in Q2 and Q4 quadrants. Images of non transduced (BF) cells and transduced cells (FITC) were performed at 10x magnification (panel (c)). Statistically significant differences were analyzed between cells transduced at 37°C and cells transduced at hyperthermia conditions. Statistical significance for results of GFP+ cells were marked as asterisks (^∗∗∗∗^*p* < 0.0001).

**Figure 5:**
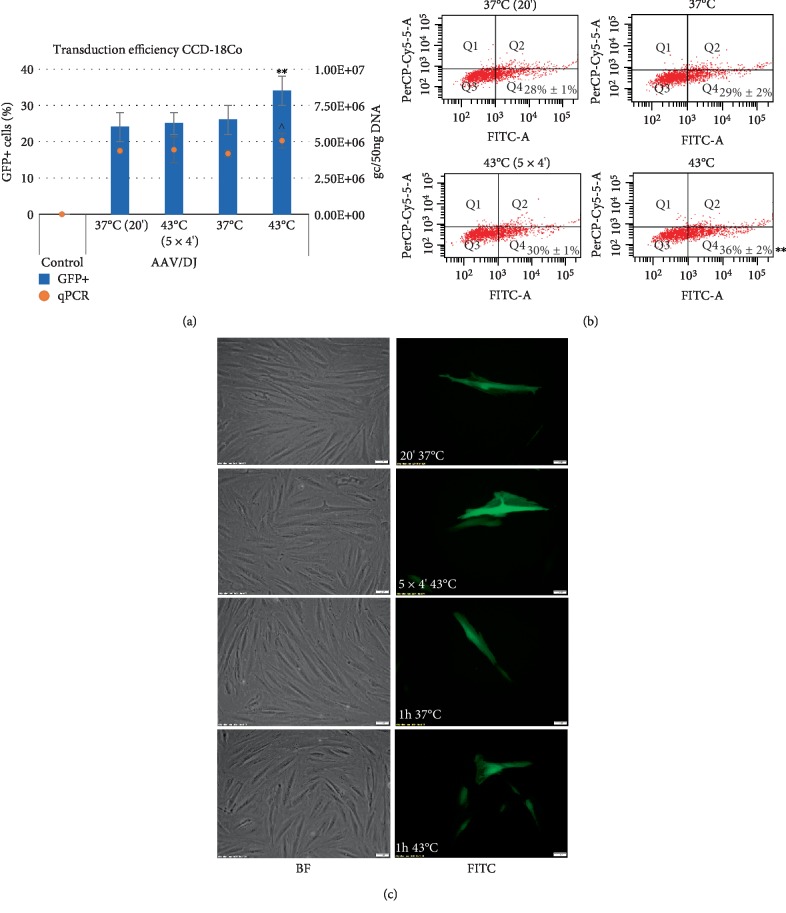
Transduction efficiency of CCD-18Co cells. The studied cells were transduced with the rAAV/DJ vector at hyperthermia conditions (as described at M&M panel). The GFP (Green Fluorescent Protein) positive (+) cells were measured by the Countess II FL Automated Cell Counter and the qPCR method (panel (a)), flow cytometry (panel (b)), and visualized with an inverted fluorescence microscope (panel (c)). For panel (b) the Q1 and Q2 quadrants represents death cells (7-AAD positive staining). The Q3 and Q4 quadrants represents live cells (7-AAD negative staining). The GFP+ cells are located in Q2 and Q4 quadrants. Images of non transduced (BF) cells and transduced cells (FITC) were performed at 10x magnification (panel (c)). Statistically significant differences were analyzed between cells transduced at 37°C and cells transduced at hyperthermia conditions. Statistical significance for results of GFP+ cells were marked as asterisks (^∗∗^*p* < 0.01) and for results of rAAV genome copies (gc) were marked as carets (^*p* < 0.05).

The efficiency of the transduction of cancer cells RKO, HT-29, LS411N, and CCD-18Co fibroblasts was evaluated by counting GFP+ cells with an automated cell counter with fluorescence detection, flow cytometer, and by assessment of the rAAV copy number with qPCR. The results are shown in the Figures 2–5. To analyze expression of GFP, the cells were harvested 6 days after transduction. As shown in Figures 2–5, the exposure of CRC cells to elevated temperature (43°C, hyperthermia) results in an increase in the rAAV transduction efficiency. The examined cells under hyperthermia are transduced at a higher level than in the normal conditions at 37°C. The tests performed with an automated fluorescence cell counter and FACS, respectively, showed that transduction efficiency in the elevated temperature reached 40%/51% for RKO, 30%/29% for HT-29, 10%/6% for LS411N, and 34%/36% for CCD-18Co. The viability of cells subjected to heat shock was determined as 95–98%. Under basic conditions (37°C) cells transduce at a lower level. In the study the sequential exposure of cells to hyperthermia was also performed. A cycle with five 4-minute temperature shocks (43°C) was used. As shown in Figures 2–5, the procedure did not reveal significant differences in the number of GFP+ cells, excluding LS411N cells (an increase from 7% to 11% GFP+; Figure 4). The copy number of the rAAV/DJ vectors, assessed by the qPCR method, provides interesting information about the efficiency/mechanisms of CRC cell transduction at hyperthermia. Generally, the number of determined rAAV genome copies (gc) corresponds to the transduction efficiency and the number of GFP+ cells (Figures 2–5). It is intriguing that in e.g. the LS411N line with lower transduction efficiency (12% GFP+) a relatively high rAAV copy number (5.3E + 06 gc) was shown. A similar copy number was obtained for the HT-29 line (6.4E + 06) but in that case, transduction efficiency was estimated at 30% GFP+. Equally, for the CCD-18Co line the number of transduced cells (34% GFP+) corresponds to 5.1E + 06 gc of rAAV. However, RKO cells transduced at the level of 40% (40% GFP+) with the rAAV copy number being estimated at only 1.2E + 05 gc. The obtained results suggest the possibility of different mechanisms of transmission/metabolism of rAAV.

### 3.2. Expression of rAAV Receptors at Hyperthermia Conditions

**Figure 6:**
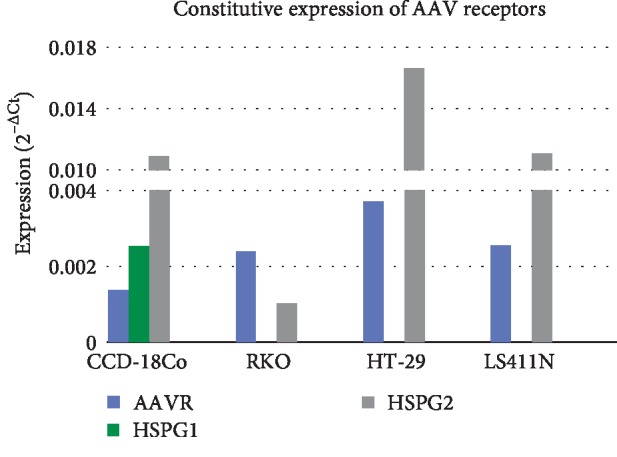
AAVR, HSPG1, HSPG2 expression at normal temperature (37°C) in RKO, HT-29, LS411N, and CCD-18Co fibroblast. The figure shows relevant differences between constitutive expression of tested genes (2^−ΔCt^).

**Figure 7:**
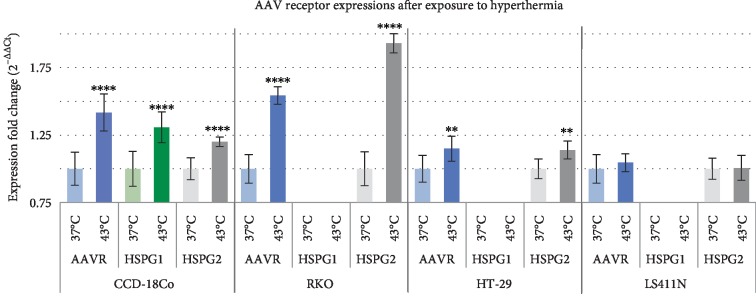
AAVR, HSPG1 and HSPG2 expression in CRC lines. The influence of heat shock on the expression of genes in tested cells as well normal cells was estimated by the reverse transcription and the qPCR method. The cells not exposure to hyperemia were used as reference to calculate fold range (2^−ΔΔCt^). Statistically significant differences were analyzed between cells incubated at 37°C and cells exposed to hyperthermia conditions (^∗∗^*p* < 0.01; ^∗∗∗^*p* < 0.0001).

The expression analysis of representative AAV receptor genes (AAVR, HSPG1, HSPG2) was performed with the qPCR method with the use of TaqMan probes. As shown in Figures 6 and 7, the examined cell lines are characterized by a different expression pattern of the rAAV transmission genes in both constitutive (37°C) and hyperthermia (43°C) conditions. In the context of the search for mechanisms to increase the number of GFP+ cells after exposure to heat shock, it is interesting that the expression of AAV receptors was changed in the CRC cells in response to elevated temperature. Outstanding results were obtained especially for AAVR and HSPG2 genes. As shown in Figure 2, the RKO cells are most efficiently transduced rAAV at hyperthermia (40% GFP+) which correspond to the highest increase in the expression of AAVR and HSPG2 genes. The upregulation of AAVR and HSPG2 expression was also observed for the HT-29 and LS411N cell lines, but at a lower level which may correspond to lower transduction efficiency, 30% GFP+ and 10% GFP+, respectively. Figures 6 and 7 also present the expression of AAV receptors in CCD-18Co control cells. The results show that CCD-18Co fibroblasts have a specific gene signature of AAV receptors that is different than in CRC cells. The presence of HSPG1 gene expression is characteristic. CCD-18Co cells respond to hyperthermia with increase in expression of all three AAV receptors.

### 3.3. HSP Expression at Normal and Hyperthermia Conditions

**Figure 8:**
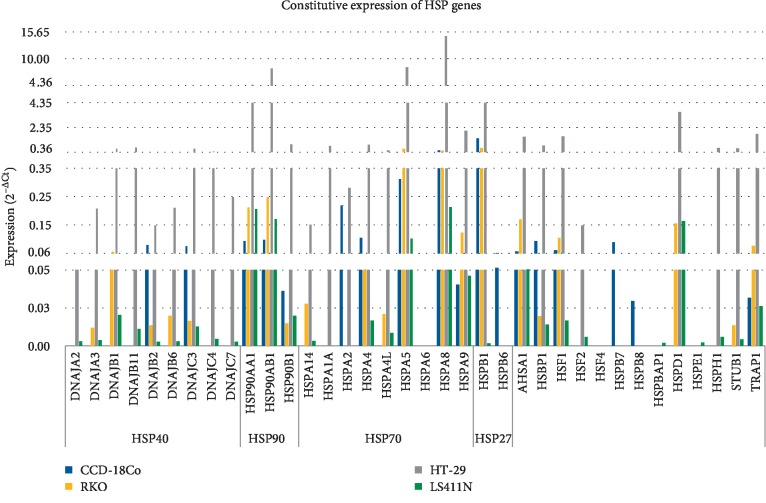
Heat shock protein (HSP) expression profiles of CRC cell lines (RKO, HT-29, LS411N) and CCD-18Co at normal temperature (37°C). The figure shows relevant differences between constitutive expression of HSP (2^−ΔCt^).

**Figure 9:**
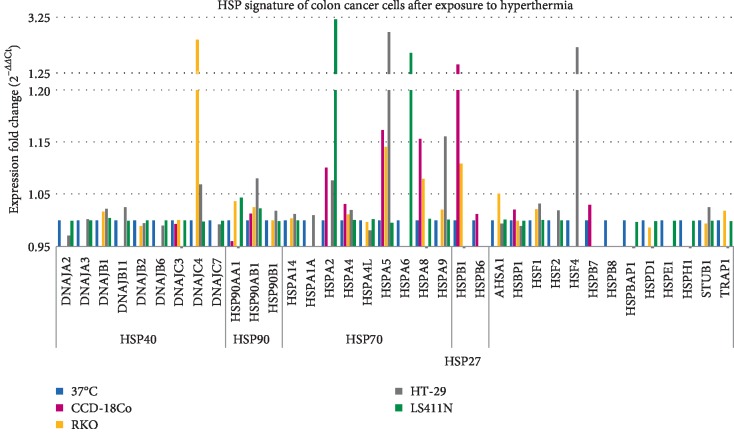
Temperature-related heat shock proteins (HSP) expression profiles of CRC and fibroblast of colon. The cells were exposed to hyperthermia (43°C, 1 h). The heat shock conditions reveal the differences between the sensitivity of CRC cells to increased temperature. Expression values (2^−ΔΔCt^) illustrate the specific HSP signature for CRC of different origin. Samples at 37°C were not exposed to hyperthermia conditions and were used as reference to calculated fold change (2^−ΔΔCt^). Expression in 37°C was marked as 1.0 for each gene, regardless of whether it was determined in all examined cells (exact results of HSP expression at 37°C were showed in Figure 10).

**Figure 10:**
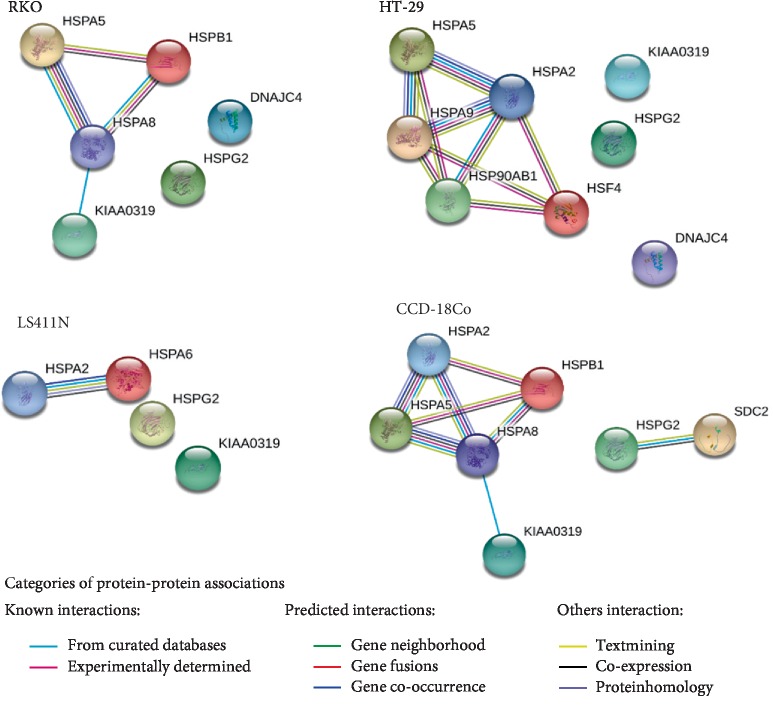
Networks of upregulated genes after hyperthermia was applied to CRC cell lines and colon fibroblast (based on data from Table 1). The interactions were visualized as nodes and colored edges (according to String database [38]).

Analysis of HSP gene expression was performed with the use of TaqMan Array Human Heat Shock Proteins. Expression levels of 42 HSP genes representing key members of HSP27, HSP40, HSP70, HSP90 families were assessed. The constitutive expression (37°C) and expression after exposure to hyperthermia (43°C) were evaluated. The results are shown in Figures 8 and 9. The potential functional relationships between HSP genes (expressed at high levels) were also presented as string diagrams (Figure 10). The results indicate that the investigated RKO, HT-29, and LS411N cell lines express HSP genes at different levels under both 37°C (Figure 8) and 43°C (Figure 9). Under the hyperthermia conditions cells respond with visible changes in HSP expression. Based on the obtained results, it is possible to propose specific HSP expression signatures characteristic for the tested CRC lines and potentially responsible for rAAV transduction under hyperthermia. The cells most efficiently transduced with rAAV, RKO (40% GFP+) after exposure to hyperthermia are characterized mainly by an increase in the levels of DNAJC4, HSPA5, HSPA8, and HSPB1. HT-29 cells (30% GFP+) are characterized especially by increased levels of DNAJC4, HSP90AB1, HSPA2, HSPA5, HSPA9, and HSF4 upon exposure to hyperthermia. LS411N cells with the lowest efficiency of rAAV transduction (10% GFP+) after exposure to hyperthermia are characterized mostly by HSPA2 and HSPA6. Control CCD-18Co cells (34% GFP) are characterized in 43°C mainly by expression of HSPA2, HSPA5, HSPA8, and HSPB1. The obtained HSP expression signatures confirm the information that CRC are not molecularly a homogeneous group of neoplastic diseases; therefore, they may respond differently to temperature shock. The results allow a better understanding of the mechanisms of rAAV transduction under hyperthermia. Potential relationships between rAAV transduction, AAV receptor, and HSP expression are collected in Table 1.

## 4. Discussion

In the presence of the serious global data on the incidence and mortality of CRC, the need for research of new treatment solutions is obvious and extremely urgent. The formula of personalized medicine is particularly visible and up-to-date in CRC. Personalized medicine considers the potential of ultrasensitive medical diagnosis methods as well as the presence of targeted medications. It appears that in the beginning gene therapy was based on primarily treating the causes of diseases and perfectly fulfills the requirements of personalized medicine. Over two thousand clinical trials of gene therapy have been performed in the world, including almost 240 involving rAAV vectors [39]. It is also known that gene preparations are included in classic oncological treatment protocols [31, 40]. The therapeutic effectiveness of gene therapy is inextricably linked to the efficiency of gene transfer to target cells. The type of vector used, the vector titer (dose), and extracellular and intracellular transduction barriers influence the gene transfer [41, 42].

In the current study, attention was paid to the possibility of increasing the transduction efficiency with rAAV vectors to CRC cells by hyperthermia. Our tests were performed based on the rAAV mosaic vector (rAAV/DJ), which expresses GFP under a strong CAG promoter. As shown in Figures 2–5, elevated temperature conditions increase the efficiency of rAAV transduction. The percentage of GFP+ cells is higher in hyperthermia conditions. In general, the stimulating effect of elevated temperature on rAAV transduction efficiency was observed for all three CRC tested (RKO, HT-29, LS411N). Differences between percentages of GFP+ cells between lines were observed. The most efficiently transduced in hyperthermia were RKO (40%), then HT-29 (30%) and LS411N has the lowest level (10%). The study also evaluated the level of rAAV/DJ genome copies in transduced cells. Due to the high transduction efficiency of RKO cells (40% GFP+) with a simultaneously low rAAV number (1.2E + 05 gc), it can be speculated that hyperthermia involves potential intracellular rAAV degradation mechanisms in CRC cells. For LS411N cells, however, an inverse effect was observed, i.e., with a low GFP+ cell count (10%) the number of rAAV copies was relatively high (5.3E + 06 gc) suggesting that LS411N cells are more resistant to potential rAAV degradation stimulated by hyperthermia. On the other hand, HT-29 cell line transduced in 30% yield (GFP+), and the rAAV copy number was estimated at 6.4E + 06 gc (Figure 3). The increase of rAAV transduction efficiency in response to the increased temperature was also demonstrated in the work of other authors. The publication of Zhong et al. [21] showed that 4 h exposure of Hela cells to a temperature of 42.5°C caused about 6x increase in rAAV transduction efficiency. In this study, 1 h stimulation at 43°C was used in different combinations. The cells were subjected to one-time 1 h exposure to 43°C or a thermal shock procedure was applied (see M&M). However, the mentioned shock cycle method, as shown in Figures 2–5, do not introduce significant differences in the number of GFP+ cells.

Looking at the potential mechanisms of the increase in the number of GFP+ cells, the obtained results were analyzed in relation to the evaluated expression of AAV receptor and HSP genes (Table 1). The signatures of both AAV receptor genes and HSP are different for the tested CRC lines. String diagrams (Figure 10) show the possible functional associations between the proteins of the examined transcripts (HSP and AAV receptors). The obtained results show hyperthermia induces changes in the expression of selected genes. It can be postulated that the assessed transcript signatures determine the reaction of CRC cells to rAAV under hyperthermia. The gene expression patterns of the tested cells (AAV receptors and HSP) correspond to the increase of rAAV transduction at elevated temperatures. As shown in Figure 7, in cells stimulated under hyperthermia, the expression of AAVR and HSPG2 genes increased predominantly. In the presence of the recently described key role of AAVR in the transduction of rAAV [43], the obtained results can be considered as interesting information. The increase in the number of GFP+ cells observed at an elevated temperature is in relation to significant contribution in the rAAV cellular transmission of the transmembrane protein (KIAA0319L) defined as AAVR, demonstrated by Pillay et al. [44–46].

In rAAV transduction cells, the role of immunophilin proteins is indicated. Immunophilins belong to highly conserved immunosuppressive drug-binding proteins, such as FK506 (tacroimus). FK506-binding proteins (FKBPs) are representative proteins for immunophilins. FKBPs act as cellular chaperones [47, 48]. An example of a protein that acts as a chaperone is FKBP52. Qing et al. [23] showed that the adeno-associated virus D-sequence-binding protein is a FKBP52. Subsequent work clarified the role of FKBP52 in the transduction of rAAV by determining the contribution of HSP [21, 23]. The works of Zhong et al. [21] and Zhao et al. [22] showed to increase the efficiency of rAAV transduction at a higher temperature in connection with the expression of selected HSP and FKBP52 genes. It was observed that phosphorylated FKBP52 interacts with D-sequences in inverted terminal repeats of adeno-associated virus 2 genome, which consequently leads to inhibition of the second strand of AAV DNA synthesis, and further to inefficient expression of reporter transgenes, i.e. a decrease in transduction efficiency.

In the present study a broader assessment of the HSP gene expression in cells exposed to elevated temperature in relation to the transduction efficiency of rAAV was attempted. A panel of 42 transcripts of HSP mRNAs representing the key HSP families HSP27, HSP40, HSP70, HSP90 was evaluated (Figure 8). Differences in constitutive as well as heat-stimulated HSP expression were demonstrated. During analysis of HSP profiles, we concentrated on proteins potentially involved in the interaction with FKBP and thereby involved in the transduction/expression of rAAV. As reported in the other works [21, 22], the efficiency of tumor cell transduction by rAAV can be limited by the expression of FKBP52 and interactions with HSP90. Furthermore, we paid attention to HSPA2 and HSPA6 in obtained HSP profile, due to the defined interactions with FKBP6 (FKBP36), chaperone protein in the immunophilin family [49]. As shown in Figure 9, CRC cells have different levels of HSPA2 and HSPA6, which may be in relation to the level of transduction at an elevated temperature and the potentially low number of rAAV gene copies in RKO cells (Figure 2). In the context of understanding the mechanisms of viral vector transduction into cells, it is interesting to notice that silencing of the FKBP6 gene enhances HIV-1 replication in HeLa-CD4 cells infected with viral pseudotypes HIV89.6R and HIV8.2N [50]. It can be speculated that the potential mechanism of FKBP6 participation in rAAV transduction may be comparable to that described for HIV-1. FKBP6 can also interact with clathrin and HSP72, HSP90AA, HSP90AA5P, and HSP90AB1 [51, 52].

Due to the participation of HSP in the development of cancers, HSP inhibitors are investigated as new factors supporting anti-cancer therapies [13]. Based on the information on the influence of HSP in the vector transfer to cells in gene therapy, it is possible to see the application of HSP inhibitors in enhancing the efficiency of cancer gene therapy. Similarly, when looking for mechanisms regulating the transfer of rAAV, attention is also paid to contribution in the rAAV transduction of the small proteins termed as SUMO and SUMOylation pathways. Holscher et al. [53] indicate the participation of SUMOylation in the transduction of rAAV. According to the authors, modulation of SUMOylation by interacting with the main system proteins (E1 conjugation enzyme Ubc9 and E3 enzymes) may increase the efficiency of transduction of rAAV. It is also indicated that HSP, e.g., HSP27, may be involved in modifications of other proteins with the participation of SUMO-2/3 [54], which may suggest a significant contribution of HSP-SUMO pathways in the transduction of rAAV.

Hyperthermia is a method of increasing the effectiveness of therapeutic protocols used in oncology [10]. Current nanomedicine is based on the use of recombinant viral particles or vectors commonly used in gene therapy strategies. The attractive biochemical-physical characteristics of rAAV, like small size, organ tropism, and nonpathogenicity [55], favor the introduction of rAAV for the treatment. Glybera and Luxturna are examples of commercialized gene therapeutics [56]. The current challenge in the field of rAAV vectorology is directly linked to increasing the efficiency of cell transduction. Efficient gene transfer to CRC cells would allow a combination of classic protocols for oncological treatment such as hyperthermic intraperitoneal chemotherapy (HIPEC) with gene therapy.

## 5. Conclusion

The present study suggests that increased rAAV transfer to CRC cells can be obtained under elevated temperature conditions. Finally, we postulate that the response of CRC cells to rAAV in hyperthermia is related with specific AAVR and HSP gene signatures.

## Figures and Tables

**Figure 1:**
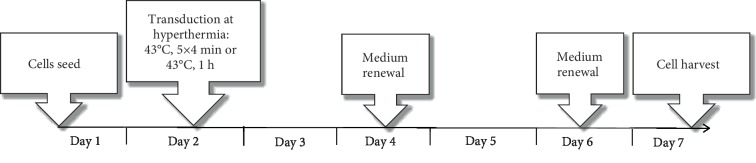
Schematic methodology of rAAV transduction at hyperthermia. The key points of the experiment were marked and described on the timeline. The rAAV vector in the dose of 4 × 10^4^ gc was added on the second day of the experiment. The CRC cells and colon fibroblasts were transduced for 48 h, then the media were replaced. The transduction efficiency was performed on seventh day of the experiment by GFP+ cells counting and qPCR method.

**Table 1:** Summary of hyperthermia influence on rAAV transduction efficiency of CRC and colon fibroblast cells.

CRC lines			RKO	HT-29	LS411N	CCD-18Co
Hyperthermia conditions (43°C, 1 h)	rAAV transduction efficiency		40%	30%	10%	34%
	Increase of rAAV receptors expression	AAVR	↑↑↑	↑↑	↑↑	↑↑
		HSPG1	-	-	-	↑↑
		HSPG2	↑↑	↑↑	↑	↑↑
	Increase of HSP expression	DNAJC4	↑↑↑↑	↑↑		
		HSP90AB1		↑↑		
		HSPA2		↑↑	↑↑↑↑	↑↑
		HSPA5	↑↑	↑↑↑		↑↑
		HSPA6			↑↑↑↑	
		HSPA8	↑↑			↑↑
		HSPA9		↑↑↑		
		HSPB1	↑↑			↑↑↑↑
		HSF4		↑↑↑↑		

The table collects the key results from Figures 7 and 9. The levels of upregulated genes after exposed to hyperthermia were marked as arrows. Expression of rAAV receptors: ↑ 0.5–1.0; ↑↑ 1.01–1.5; ↑↑↑ 1.51–2.0; and expression of HSP: ↑ 0.95–1.05; ↑↑ 1.051–1.15; ↑↑↑ 1.151–1.20; ↑↑↑↑ 1.25–3.25. The analyzed results were expressed in the form of 2^−ΔΔCt^ (expression fold change).

## Data Availability

The datasets used to support the findings of the current study are available from the corresponding author upon reasonable request.
